# Identification of Duplication Genotypes of the Feathering Rate Gene in Chicken by a Multiplex PCR Following Electrophoresis and/or Sanger Sequencing

**DOI:** 10.3390/ani13061091

**Published:** 2023-03-18

**Authors:** Qingmiao Shen, Junying Li, Haigang Bao, Changxin Wu

**Affiliations:** National Engineering Laboratory for Animal Breeding, Beijing Key Laboratory of Animal Genetic Improvement, College of Animal Science and Technology, China Agricultural University, Beijing 100193, China

**Keywords:** chicken, feathering rate, tandem duplication, multiplex PCR

## Abstract

**Simple Summary:**

Autosexing based on sex-linked phenotypes of late feathering (LF) and early feathering (EF) was widely used in poultry production. Previous studies found that tandem duplication on the Z chromosome is responsible for LF. In this study, based on tandem duplication, we developed a multiplex PCR test for genotyping through reaction optimization. The genotyping result for feathering rate with the multiplex PCR test was consistent with the sex and phenotype records, and the result of homozygote and heterozygote tests of LF males were verified by test cross and tallied with offspring’s phenotypes. The multiplex PCR test is stable and accurate for feathering rate genotyping, applicable to all LF chickens associated with tandem duplication, and has great significance for poultry breeding.

**Abstract:**

Sex-linked phenotypes of late feathering (LF) and early feathering (EF) are controlled by a pair of alleles *K* and *k^+^*. Autosexing based on the feathering rate is widely used in poultry production. It is reported that a tandem duplication of 176,324 base pairs linked to the *K* locus is responsible for LF expression and could be used as a molecular marker to detect LF chicken. So far, there is no genotyping method that can accurately and stably identify the LF homozygote and heterozygote in all chicken breeds. In the present study, a multiplex PCR test was developed to identify EF, LF homozygote, and heterozygote according to electrophoretic bands and the relative height of the peaks by Sanger sequencing. We tested 413 chickens of six native Chinese breeds with this method. The identification was consistent with the sex and phenotype records of the chickens. Band density analysis was performed, and the results supported our genotyping using the new assay. In order to further verify the accuracy of this test in distinguishing homozygote and heterozygote males, 152 LF males were mated with EF females, and the results of the offspring’s phenotypes were consistent with our expectations. Our results support tandem duplication as molecular markers of LF, and this new test is applicable to all LF chickens associated with tandem duplication.

## 1. Introduction

Sex identification of newly hatched chicks is an important part of the poultry industry, which directly affects production and economic value. Autosexing based on sex-linked phenotypic features (plumage color and pattern, feathering rate, etc.) can achieve nearly 100% sex identification accuracy [1]. Compared to a direct check of the presence or absence of rudimentary phallus in the ventral part of the cloaca to identify sex, autosexing can not only avoid injury to chicks but also be more convenient and accurate [2]. So, it was widely used by hatcheries. Sex-linked phenotypes of late feathering (LF) and early feathering (EF) are controlled by a pair of alleles of *K* and *k^+^* in Chromosome Z. The distinction between EF and LF is based on the length of the primary feathers and primary-convert feathers at hatching, with primary feathers more than 2 mm longer than primary-convert feathers being EF, otherwise, they are considered as LF [3]. *K* allele is incompletely dominant to *k^+^* and has a retard effect on the primary and secondary flight feathers growth [4,5,6,7]. When homozygous EF males (*k^+^/k^+^*) mate with LF (*K/−*) females, their progeny males are LF (*K/k^+^*), and females are EF (*k^+^/−*), thus achieving gender separation. For poultry breeders, the establishment of EF and LF pure-breeding strains is the key to autosexing. Pure-breeding EF strain can be selected directly in one generation based on phenotypes, while it takes at least two generations to establish a pure-breeding LF strain because the homozygote and heterozygote roosters are generally identified by test cross. Therefore, it is of great significance to the poultry industry to explore suitable molecular markers and effective methods to distinguish LF homozygote and heterozygote, which can not only avoid test cross but also save the economic and time costs of establishing Pure-breeding LF strain.

Previous studies found that the *K* locus is tightly linked to endogenous retrovirus 21 (*ev21*) fragment and has a negative effect on production performance [8,9,10]. The genetic distance of the *K* locus and *ev21* is less than 0.3 cM apart [11]. So, several pieces of research have been developed to distinguish EF and LF based on the molecular marker of *ev21* [12,13,14,15]. However, *ev21* was found in some EF breeds and lacked in some LF breeds, which revealed that *ev21* is not responsible for the expression of LF and is not an optimal molecular marker [13,16,17,18]. Elferink et al. reported that the molecular architecture of the *K* locus, tandem duplication of 176,324 base pairs at the *K* allele, consists of the partial duplication of the prolactin receptor (*PRLR*) and sperm flagellar protein 2 (*SPEF2*) [19]. Bu et al. and Zhao et al. suggested that the partial duplication of *PRLR* and *SPEF2* may be the cause of LF expression [20,21,22]. Takenouchi et al. checked the tandem duplication in over 50 chicken breeds and indicated that tandem duplication is responsible for LF expression. Therefore, it should be used as a molecular marker to detect LF birds [18].

Based on the molecular marker of tandem duplication, a few qRT-PCR and PCR-RFLP tests have been developed to distinguish homozygote and heterozygote cocks. However, the stability and accuracy of qRT-PCR tests could be improved, and PCR-RFLP tests might not be applicable to all chicken breeds [19,23,24]. More effective and accurate tests need to be explored. Multiplex PCR is a technology that simultaneously amplifies two or more targets through one reaction, including more than one pair of primers, thereby realizing the diagnosis of multiple targets [25]. As a firmly established general technique, multiplex PCR has been widely used in pathogen identification, genotyping, quantitative genetic disease diagnosis, and other fields [26,27,28,29,30]. In this study, we checked the molecular marker of tandem duplication in six chicken breeds and developed a multiplex PCR test to identify EF, LF homozygote, and heterozygote chickens.

## 2. Materials and Methods

### 2.1. Chickens and DNA Collection

All 413 chickens, including 228 LF chickens and 185 EF chickens, we used in this study were reared at the experimental base of the College of Animal Science and Technology, China Agricultural University, and listed in Table 1. We recorded the EF and LF phenotypes of all individuals after they hatched. A blood sample of each chicken was collected from the wing vein using 1 mL injectors at 30–35 weeks. Genomic DNA was extracted from all blood samples using the TIANamp Genomic DNA Kit (Cat. #DP304-03, TIANGEN Biotech (Beijing) Co., Ltd., Beijing, China) according to the manufacturer’s instructions. DNA quality and concentration were measured by NanoDrop 2000 Spectrophotometer (Thermo Fisher Scientific Inc., Waltham, MA, USA).

### 2.2. Primers

**Figure 1:**
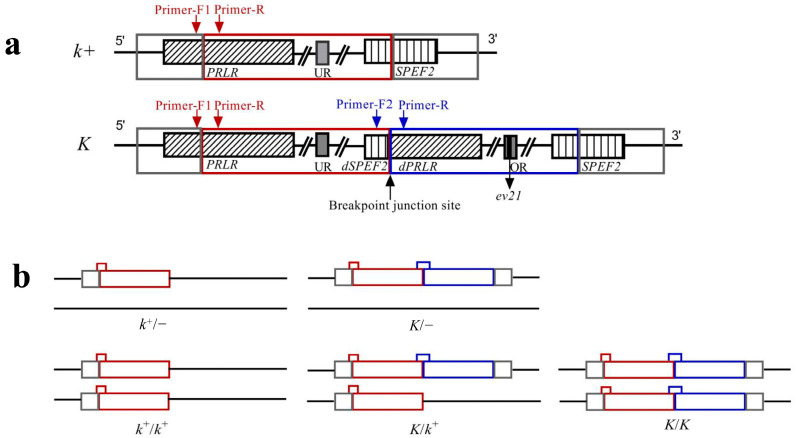
The positions of primers on *K* and *k^+^* alleles. (**a**) The red and blue boxes indicate the tandem duplication block; UR indicates the unoccupied *ev21* integration region; OR indicates the occupied *ev21* integration region; Primer positions are indicated by red and blue arrows. (**b**) Fragment regions that can be amplified by multiplex PCR with DNA samples from different feathering chickens. The small red box represents the 386 bp fragment amplified by the primer F1-R, and the small blue box represents the 786 bp fragment amplified by the primer F2-R. Within DNA samples, the ratio of the number of 786 bp and 386 bp templates of heterozygote (*K*/*k^+^*) and homozygote (*K*/*K*) is 0.5 and 1.0, respectively. Under the same total DNA concentration, the number of *K* alleles of LF homozygote (*K*/*K*) is quite different from that of LF female (*K*/*−*), and their ratio is 2:1.

Based on the tandem duplication, primers’ locations for multiplex PCR are shown in Figure 1a [19]. The primers were designed using Primer 5.0 software (PREMIER Biosoft, San Francisco, CA, USA) according to the sequence information of the GRCg7b genome(Chromosome Z, NC_052572.1). Primer sequences are listed in Table 2. The 786 bp multiplex PCR product spans the breakpoint junction. If the tandem duplication is present in chickens, the 786 bp and 386 bp products both can be amplified. Otherwise, only the 386 bp product was amplified. The products that can be amplified by multiplex PCR in different feathering are shown in Figure 1b. In theory, the brightness of the agarose gel electrophoretic bands of products should be consistent with the dosage effect.

### 2.3. Optimization of Multiplex PCR Conditions

The multiplex PCR reaction was performed in a 20 µL mixture system, containing 10 µL of 2 × Taq PCR Mix (Beijing HT-biotech Co., Ltd., Beijing, China), 0.25 µL of the F1 primer, 0.5 µL of the R primer, 0.25 µL of the F2 primer (The concentration of each primer is 10 pmoL/µL), 1 µL of DNA template and 8 µL of ddH_2_O. For multiplex PCR, annealing temperature (Ta), cycle number, and nucleotide concentration are very critical factors, so one LF female, one LF homozygous male, and one LF heterozygous male with known genotypes were used to optimize the reaction conditions in order to obtain the optimum conditions. First, the optimal annealing Ta value of the primers was tested by a gradient PCR with an increasing annealing Ta from 60 °C to 70 °C on a Biometra TOne thermal cycler(Analytik Jena AG, Jena, Germany). The thermal cycling process was as follows: 95 °C for 5 min, 28 cycles of denaturation at 95 °C for 30 s, annealing Ta for 30 s, and extension at 72 °C for 50 s, with a final extension at 72 °C for 5 min. Six µL of PCR product and DL2000 DNA Marker (Tsingke Biotechnology Co., Ltd., Beijing, China) were separated on a 1.5% agarose gel treated with SYBRTM Safe DNA Gel Stain (Thermo Fisher Scientific Inc., Carlsbad, CA, USA) at 120 V for 30 min. Electrophoretic bands were visualized using G:Bax Instrument (Synoptics Ltd., Cambridge, UK), and the optimal Ta was obtained based on the results of electrophoretic bands.

Under the optimal Ta, we tested the influence of cycle number N (26, 28, 30, 32) and DNA template concentration (40 ng/µL, 60 ng/µL, 80 ng/µL, 100 ng/µL) on the PCR reaction. The PCR reaction system, thermal cycling process, and agarose gel electrophoresis were described above, except for the number of cycles and the DNA template concentration. ImageJ software (version 1.53t; National Institutes of Health, Bethesda, MD, USA) was used to calculate the band density of the electrophoretic bands. After electrophoresis, the remaining PCR products were subjected to Tsingke Biotechnology Co., Ltd. (Beijing, China) for Sanger sequencing with R primer.

### 2.4. Genotyping and Verification of LF Males by Test Cross

Based on the PCR optimization test, the optimal conditions for genotype identification were obtained. The PCR reaction system, thermal cycling process, agarose gel electrophoresis, and Sanger sequencing were described as optimization tests. We identified EF and LF chickens based on the number of electrophoretic bands. EF only has a 386 bp band, and LF has a 386 bp band and 786 bp band. LF homozygote and heterozygote were identified according to the relative brightness of the two bands and the Sanger sequencing peaks. Electrophoretic band density of all 228 LF chicken were determined using ImageJ software (version 1.53t; National Institutes of Health, Bethesda, MD, USA) and adjusted by the band density of the 750 bp band of DL2000 DNA Marker (Tsingke Biotechnology Co., Ltd., Beijing, China). The ratio and sum of band density of 786 bp and 386 bp bands of each sample were calculated. Within DNA samples, the ratio of the number of 786 bp and 386 bp templates of heterozygote *(K*/*k^+^)* and homozygote (*K*/*K*) is 0.5 and 1.0, respectively (Figure 1b). Accordingly, under the optimized PCR amplification condition, the ratio of 386 bp and 786 bp bands amplified by LF homozygote and heterozygote samples may also be obviously different. Here, the parameter of the ratio of band density of 786 bp and 386 bp bands was used to distinguish LF homozygote and heterozygote. Under the same total DNA concentration, the number of *K* alleles of LF homozygote (*K*/*K*) is quite different from that of LF female (*K*/*−*) (Figure 1b). Accordingly, we used a parameter of the sum of band density of 786 bp and 386 bp bands corrected by Marker to distinguish LF homozygote and LF females.

After genotyping all samples by multiplex PCR following electrophoresis and Sanger sequencing, we verified the LF homozygote and heterozygote by test cross with three or more EF females of an EF chicken strain. If an LF rooster has more than 5 LF and no EF newly hatched offspring, the rooster is considered a homozygote. An LF rooster is considered a heterozygote if it has one or more EF offspring.

## 3. Results

### 3.1. Optimization of Multiplex PCR Conditions

**Figure 2:**
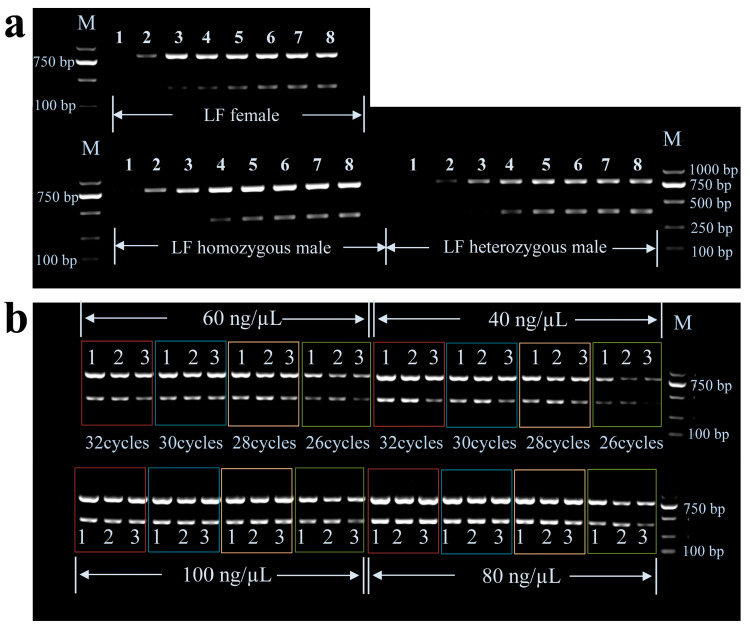
The agarose gel electrophoretic bands of multiplex PCR optimization test. M represent DL2000 DNA Marker. (**a**) The multiplex PCR agarose gel electrophoretic bands of different Ta. 1–8 represent 69.9 °C, 68.7 °C, 67.0 °C, 65.0 °C, 63.3 °C, 62.1 °C, 61.2 °C, 60.6 °C, respectively. (**b**) The multiplex PCR agarose gel electrophoretic bands of different cycle numbers and DNA template concentration; 1, 2, and 3 represent LF homozygous males, LF heterozygous males, and LF females, respectively.

One LF female, one LF homozygous male, and one LF heterozygous male with known genotypes were used to optimize the multiplex PCR reaction conditions. By a gradient multiplex PCR with an increasing annealing Ta from 60 °C to 70 °C and agarose gel electrophoresis, the optimal annealing temperature value of the primers was determined as 60.6–63.3 °C (Figure 2a). We optimize the number of reaction cycles and DNA template concentration for the multiplex PCR at 61 °C. The multiplex PCR electrophoretic bands of different cycle numbers N (26, 28, 30, 32) and different DNA template concentrations (40 ng/µL, 60 ng/µL, 80 ng/µL, 100 ng/µL) were shown in Figure 2b, and band density were shown in Table S1. From the two bands' brightness of Figure 2b, we can see that at 26 cycles, 40–100 ng/µL DNA template concentrations can be used to identify homozygote and heterozygote; at 28 cycles, 40–60 ng/µL DNA template concentrations can be used to identify; at 30 and 32 cycles, none of the four DNA template concentrations seemed to identify genotypes. From Table S1, we can see that the ratio of 786 bp band density to 386 bp band density is more different between homozygote and heterozygote at low concentration and low cycle. Therefore, we suggested the optimal conditions of multiplex PCR for genotyping were set with a Ta value of 61 °C, 26 cycles, and lower DNA template concentration.

**Figure 3:**
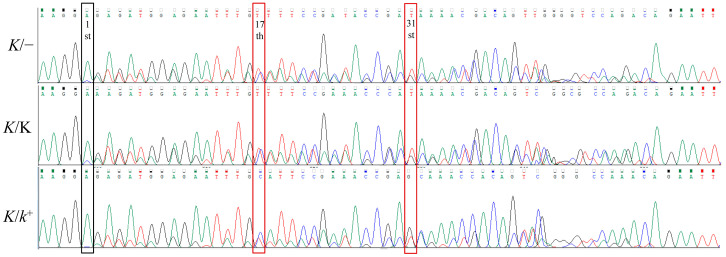
The part of the Sequencing peak of LF by Sanger sequencing with R primers. After passing the breakpoint, there is a section of double peaks in LF. The first doublet peak after the breakpoint is counted as the 1st, and the peaks of the 17th and 31st positions always show obvious changes in LF female, LF homozygote, and heterozygote. These two positions could be taken as two molecular markers for homozygote and heterozygote identification.

After Sanger sequencing with R primer, part results of sequencing peaks of LF are shown in Figure 3. From primer R to the breakpoint junction site, the sequences of different genotypes are identical, so the sequencing shows a single peak. After passing the breakpoint, the amplified sequence is different, so a double peak appears until the position of primer F1, and then only a single sequence until primer F2 corresponds to a single peak. Due to the dosage effect, relative differences in the concentrations of homozygote and heterozygote PCR product bases may result in a difference in the height at double peaks. Under the 26 cycles, 40–100 ng/µL DNA template, and 28 cycles 40–60 ng/µL, the peaks of the 17th and 31st positions always show obvious changes in LF female, LF homozygote, and heterozygote. The 17th T peak height of the homozygote is equal to or higher than the C peak, and the 31st is higher than the G peak; Heterozygote shows the opposite trend to homozygote. The T peak height at the 17th and 31st are both lower than C and G peaks. Therefore, the doublet peaks at these two positions could be taken as two molecular markers for the chicken genotype.

### 3.2. Genotyping by Multiplex PCR

**Figure 4:**
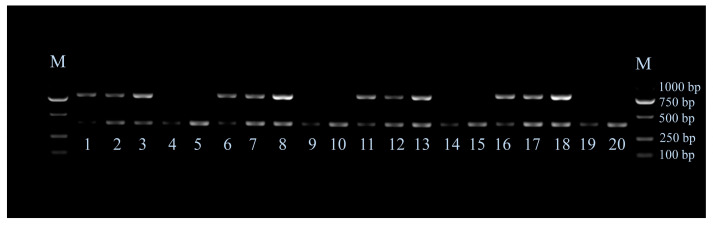
Patterns of the multiplex PCR agarose gel electrophoretic bands associated with different feathering genotypes. M represents DL2000 DNA Marker; The brightness of the bands is affected by dosage effect; 1, 6, 11, and 16 indicate *K*/−; 2, 7, 12, and 17 indicate *K*/*k*^+^; 3, 8, 13, and 18 indicate *K*/*K*; 4, 9, 14 and 19 indicate *k^+^*/−; 5, 10, 15 and 20 indicate *k^+^*/*k^+^*.

We used multiplex PCR to test the condition of Ta value of 61 °C, cycle number of 26, and the amount of DNA template of 40–50 ng in all reactions for genotyping. The agarose gel electrophoretic bands of different genotypes are shown in Figure 4. All chickens were genotyped according to the band pattern in Figure 4 and the molecular markers in Figure 3. The results revealed that all LF had tandem duplication, but all EF had none (Table 3).

The identification results of LF based on electrophoretic bands and Sanger sequencing are shown in Table S2. From Table S2, we can see that the genotyping results of electrophoresis bands are completely consistent with that of Sanger sequencing. All identification was consistent with the sex and phenotype records of the chickens.

The band density values were calculated and shown in Table S3. From Table S3, we can see that the band density ratio of 786 bp and 386 bp of each LF heterozygote is 0.61–1.51, each LF homozygote is 1.76–4.33, and each LF female is 2.03–7.51, which suggests that LF heterozygote have lower band density ratio of 786 bp and 386 bp than that of LF homozygote and LF female. After corrected by the band density of 750 bp of DL2000 DNA Marker, the sum of 786 bp and 386 bp band density of each LF homozygote is 1.41–2.69, and each LF female is 0.46–1.27. All results of the band density analysis were consistent with the results of direct eye observation according to the relative brightness of the two bands and the Sanger sequencing peaks.

### 3.3. Verifying the Accuracy of the Genotyping Result of Late Feathering Roosters by Test Cross

In the present study, a total of 152 LF males were genotyped by electrophoretic bands and Sanger sequencing. In order to verify the accuracy of the genotyping results, all males were mated with three or more EF females of an EF chicken strain. Except for one SG rooster and one LK rooster, the cross-test results of other roosters are consistent with the results of the new genotyping method. The SG and LK roosters were both identified as homozygotes by electrophoretic bands and Sanger sequencing, but each of them came up with one EF offspring during the test cross. The SG hatched out 13 LF and 1 EF offspring, and the LK hatched out 20 LF and 1 EF offspring. Details of the test cross were shown in Table S4. We then genotyped the two incongruent EF offsprings by our multiplex PCR test following electrophoretic bands and Sanger sequencing and found no tandem duplication in the two chicks, which indicated that the two chicks were not the offspring of the two LF homozygote roosters. Therefore, we speculated that the inconsistency of the two EF chicks is caused by mistakes during collecting breeding eggs or other operations rather than by genotyping errors from our new method. The results of the offspring’s phenotypes were consistent with our expectations.

## 4. Discussion

The balance of relative primer concentrations at the various loci, annealing Ta, DNA template concentration, and cycle number, among others, are critical for a successful multiplex PCR assay [25,31,32]. In this study, the two fragments share R primers, so the amount of R primers in each PCR system is twice that of F1 and F2 primers. By reaction optimization test, the multiplex PCR for genotyping was set with a Ta value of 61 °C, 26 cycles, and a DNA template concentration of 40–50 ng/µL. Below 26 cycles and 40 ng/uL due to the low concentration of the multiplex PCR product, the electrophoretic bands are less bright, which is not conducive to genotyping and may not meet the sequencing requirements when Sanger sequencing. Since the PCR instrument and Taq enzyme et al. can affect the multiple PCR amplification efficiency, the reaction conditions may be different from those in this study. Therefore, we recommend obtaining the optimal conditions by reaction optimization test. Otherwise, the accuracy of identification may be affected. The brightness of the electrophoretic bands is mainly related to the length and concentration of the product, so ensuring the total amount of DNA templates is basically equal in each reaction is the key to identification by electrophoretic bands. Based on electrophoretic bands, not only homozygote and heterozygote can be identified, but also male and female can be distinguished.

The tandem duplication has been reported to be responsible for LF expression [18,19,20,22,33]. Based on tandem duplication, qRT-PCR and PCR-RFLP tests have been developed to identify the LF homozygote and heterozygote, but there are certain limitations [19,23,24]. Elferink et al. developed a TaqMan-based quantitative PCR test to distinguish between LF homozygote and heterozygote. The accuracy rate was only 85.3% [19]. Zhang et al. used the qRT-PCR test to detect the copy number variation between candidate gene *PRLR* and reference genes *PCCA* to identify genotypes. However, both EF male and LF female contain two copy numbers, which makes it difficult to distinguish them [23]. Li et al. developed a PCR-RFLP test among 254 individuals of five chicken breeds, the genotype identification accuracy rate reached 100% [24], but this test cannot be applicable to all chicken breeds, such as Lvke and Rhode Island Red chicken. Our method relies on band intensity which may make this assay vulnerable to subjective judgment. In the present study, band density analysis was performed, and we found that there were obvious differences between LF heterozygote and LF homozygote in the ratio of band density of 786 bp and 386 bp, and there was no overlap in the range of values, while LF homozygote and LF female also have the same situation in the sum of band density of 786 bp and 386 bp corrected by Marker, which is consistent with our typing results. Therefore, we think our detection method is feasible. Compared with other methods, our new method is more accurate and can be applied to all LF chickens associated with tandem duplication.

In this multiple PCR test, the same genotyping results were obtained by electrophoretic bands and Sanger sequencing, respectively, which indicates that we can identify by either method. However, we think combining the two methods may result in more reassuring results. When the results of the two methods are different, it is recommended to redo the sample, although no such phenomenon was found in our experiment.

## 5. Conclusions

Based on the tandem duplication of the *K* locus, a new multiplex PCR test was developed to identify EF, LF homozygote, and LF heterozygote according to agarose gel electrophoretic bands and/or the relative height of the peaks by Sanger sequencing. The new method relies on band intensity which makes this assay vulnerable to subjective judgment. Band density analysis was performed, and the results supported our genotyping using the new assay. Therefore, this new test is feasible and accurate and can be applied to all LF chickens associated with the tandem duplication.

## Figures and Tables

**Table 1:** List of chicken breeds used in this study.

Breeds	Abbreviations	Phenotypes	Sex	Numbers of Chickens
Shouguang chicken	SG	LF	male	48
		EF	male	34
		LF	female	76
		EF	female	74
Beijing You chicken	BY	LF	male	59
		EF	male	6
Lvke chicken	LK	LF	male	17
Tibetan chicken	TB	LF	male	27
		EF	male	15
Huxv chicken	HX	EF	male	40
Luxi gamecocks	LXG	EF	male	16
		LF	male	1

**Table 2:** Primers are used for multiplex PCR.

Primers	Sequence(5′–3′)	Product Size
F1	GTTTGACCTGTGCTGTGGTTTGCT	F1-R:386 bp
R	CTGTGCCCTTCCATCAGTGCTTC	
F2	GCCATCAGCCAGATCCGTCAG	F2-R:786 bp

**Table 3:** The relationship between feathering rate and tandem duplication.

Breeds	Phenotypes	Sex	Numbers of Chickens	Numbers of Chickens Present of Tandem Duplication	Numbers of Chickens Absent of Tandem Duplication
SG	LF	male	48	48	
	EF	male	34		34
	LF	female	76	76	
	EF	female	74		74
BY	LF	male	59	59	
	EF	male	6		6
LK	LF	male	17	17	
TB	LF	male	27	27	
	EF	male	15		15
HX	EF	male	40		40
LXG	EF	male	16		16
	LF	male	1	1	

## Data Availability

The data presented in this study are available in Supplementary Materials.

## Supplementary Materials

The following supporting information can be downloaded at: https://www.mdpi.com/article/10.3390/ani13061091/s1, Table S1: The multiplex PCR agarose gel electrophoretic band density of different cycle numbers and DNA template concentration. Table S2: The identification results of LF chicken based on electrophoretic bands and Sanger sequencing. Table S3: The band density analysis results of LF chicken. Table S4: The test cross results of LF males.
